# Hepatic factor may not originate from hepatocytes

**DOI:** 10.3389/fcvm.2022.999315

**Published:** 2022-09-06

**Authors:** Monica Merbach, Ramani Ramchandran, Andrew D. Spearman

**Affiliations:** ^1^Division of Cardiology, Department of Pediatrics, Medical College of Wisconsin, Children's Wisconsin, Herma Heart Institute, Milwaukee, WI, United States; ^2^Cardiovascular Center, Medical College of Wisconsin, Milwaukee, WI, United States; ^3^Division of Neonatology, Department of Pediatrics, Medical College of Wisconsin, Children's Wisconsin, Milwaukee, WI, United States

**Keywords:** congenital heart disease, univentricular circulation, hepatic factor, Glenn, Fontan, pulmonary arteriovenous malformation (PAVM)

## Abstract

Pulmonary arteriovenous malformations (PAVMs) develop universally in patients with univentricular congenital heart disease. They are believed to form due to lack of an unidentified factor from hepatocytes that perfuses the lungs to maintain vascular homeostasis and prevent PAVM formation. This unidentified factor is termed hepatic factor; however, the identity, mechanism, and origin of hepatic factor are unknown. Several hepatic factor candidates have been previously proposed, but few data are available to support previous hypotheses. Recent data showed that soluble vascular endothelial growth factor receptor 1 (sVEGFR1) is enriched in hepatic vein blood and may be a potential hepatic factor candidate. We used imaging and molecular approaches with wild-type mice to determine whether sVEGFR1 originates from hepatocytes in the liver. To our surprise, we identified that sVEGFR1 is negligibly expressed by hepatocytes but is robustly expressed by the non-parenchymal cell population of the liver. This suggests that hepatic factor may not originate from hepatocytes and alternative hypotheses should be considered. We believe it is necessary to consider hepatic factor candidates more broadly to finally identify hepatic factor and develop targeted therapies for CHD-associated PAVMs.

## Introduction

Pulmonary arteriovenous malformations (PAVMs) are abnormal vascular connections that result in intrapulmonary shunting and hypoxia. PAVMs develop in 60-100% of patients following superior cavopulmonary connection (Glenn palliation) for univentricular congenital heart disease (CHD) (1, 2). Initial observations suggested that PAVMs resulted from lack of pulsatile pulmonary blood flow, lack of hepatic vein blood perfusion, or both. Greater support for the role of hepatic vein blood came from clinical cases with preferential streaming of hepatic vein blood to a single lung. Specifically, PAVMs resolve in the lung receiving hepatic vein blood and PAVMs progress in the contralateral lung (lacking hepatic vein blood perfusion). Nearly 30 years ago Dr. Richard Jonas proposed that hepatic vein blood has a critical role in preventing CHD-associated PAVMs and “absence of some important interaction between a hepatic factor and lung blood vessels induces formation of arteriovenous malformations” (3). To our knowledge, this was the first appearance of the term *hepatic factor* in the literature. Decades later, lack of hepatic factor is the generally accepted cause for CHD-associated PAVMs, yet hepatic factor remains unidentified, and our understanding has not advanced.

## Pioneering hepatic factor studies

In one of the earliest studies attempting to identify hepatic factor and its potential mechanism, Marshall et al. investigated the effect of hepatocyte-conditioned media on bovine capillary endothelial cells (4). They reported that hepatocyte-conditioned media inhibited proliferation of bovine endothelial cells and that the inhibitory activity was lost after heating the conditioned media to 95°C for 5 min. They described preliminary steps for purifying the unidentified hepatic factor with plans to sequence its amino acids (presuming that hepatic factor is a secreted protein), but no specific factors were subsequently reported. This study created the initial and still pervasive hypothesis that hepatic factor originates from hepatocytes in the liver to regulate lung endothelial homeostasis.

In addition to the hepatocyte-origin hypothesis presented by Marshall et al., they and others proposed anti-angiogenic hepatic factor candidates such as angiostatin and endostatin (4–6). These proteins were shown in other fields to have anti-angiogenic function. To our knowledge, though, they have not been studied mechanistically in PAVMs, and their precursor proteins have not been quantified in the hepatic vein and superior vena cava (SVC).

In 2013, Field-Ridley et al. investigated endostatin as a hepatic factor candidate by quantifying endostatin levels from peripheral venous blood before and after surgery in patients undergoing Glenn palliation, Fontan palliation, and a control group of biventricular repair (7). They observed that endostatin plasma levels decreased in peripheral venous blood after Glenn palliation (4.4 to 3.3 ng/ml; *p* < 0.0001) but did not change after Fontan palliation or biventricular repair. They also observed that endostatin's precursor protein (collagen XVIII) increased after Glenn palliation (optical density pre: 74,800; optimal density post: 2,148,312; *p* < 0.0001). It's unclear, though, what a decrease in peripheral vein concentration of endostatin signifies for PAVM pathophysiology.

More recently, micro RNAs (miRNAs) were hypothesized to function as hepatic factor (8). This is a novel and compelling hypothesis, but, to our knowledge, there are no published studies investigating miRNAs or other non-coding RNA in hepatic vein or SVC blood. In summary, there are multiple hypotheses about the identity, mechanism, and origin of hepatic factor but with minimal supporting data.

## More recent hepatic factor studies using paired patient blood samples

Several groups recently started analyzing paired blood samples from the hepatic vein and SVC under the premise that hepatic factor is enriched in hepatic vein blood (9–13). In one of the first publications using this approach, Capasso et al. investigated whether BMP9 and BMP10 were potential hepatic factor candidates (10). Their strong rationale was based on the known role of BMP9 and 10 as ligands for ALK1, which is associated with hereditary hemorrhagic telangiectasia (HHT). HHT is a genetic condition characterized by arteriovenous malformations in multiple vascular beds, including the lungs. They hypothesized that BMP9 and 10 may be enriched in hepatic vein blood, and lack of BMP9 and 10 perfusion to the lungs in Glenn circulation would decrease ALK1 endothelial signaling and phenocopy HHT AVM pathophysiology. Unfortunately, there were no differences in BMP9 or 10 plasma levels in the different anatomical collection sites in patients with univentricular or biventricular circulation. This suggested that BMP9 and 10 are likely not the unidentified hepatic factor.

Two additional studies independently identified that angiopoietin signaling may be a critical component of vascular remodeling in univentricular circulation (11, 12). Angiopoietin signaling is associated with vascular dysplasias, and angiopoietin-2 (ANGPT2) is a protein that promotes vascular remodeling in multiple vascular beds. These studies reported increased ANGPT2 levels in patients with Fontan (11) and Glenn circulation (12) compared to control patients with biventricular circulation. Both studies reported no difference in ANGPT2 levels between the SVC/pulmonary artery and inferior vena cava/hepatic vein. These studies proposed that ANGPT2 may play a role in vascular remodeling and clinical outcomes of patients with univentricular circulation, but ANGPT2 is unlikely to function as hepatic factor. However, other components of angiopoietin signaling (i.e., upstream regulators of ANGPT2) may still function individually or collectively as the unidentified hepatic factor.

Last, we recently identified that soluble vascular endothelial growth factor receptor 1 (sVEGFR1, also known as sFLT1) was significantly enriched in hepatic vein serum compared to SVC serum from a heterogenous cohort of children with CHD (13). Because increased VEGF signaling is associated with AVMs and supplemental sVEGFR1 can inhibit brain AVMs (14–17), we speculated that sVEGFR1 may be a potential hepatic factor candidate. Thus, as a first step to determining whether sVEGFR1 could be the unidentified hepatic factor, we investigated whether sVEGFR1 originates from hepatocytes in the liver.

## Determining the origin of hepatic vein enriched sVEGFR1

**Figure 1:**
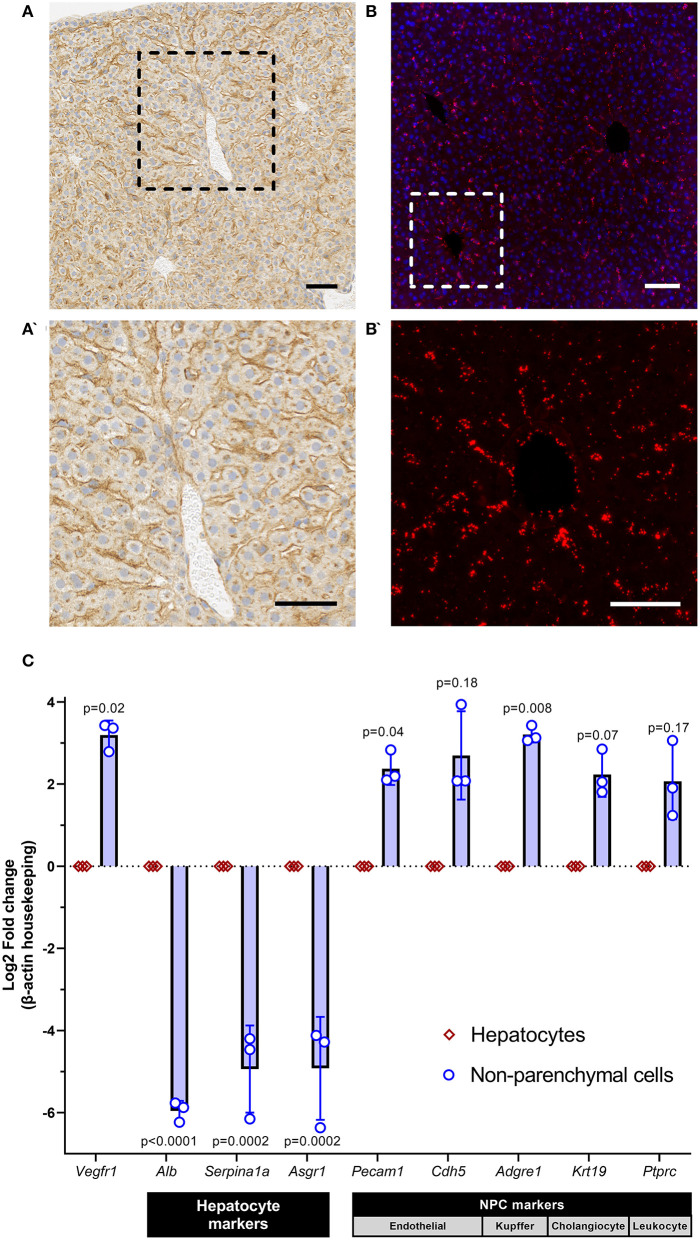
sVEGFR1 is not significantly expressed by hepatocytes in the liver. **(A,A')** Immunohistochemistry of mouse liver demonstrates strong staining in the sinusoidal regions of the liver with negligible staining in hepatocytes; scale bar 50 μm. **(B,B')** RNAscope of mouse liver demonstrates hybridization signal in the sinusoidal regions of the liver; scale bar 50 μm. **(C)** Quantitative RT-PCR of isolated hepatocytes and non-parenchymal cells (NPCs) shows significantly greater expression of *Vegfr1* in NPCs compared to hepatocytes; *n* = 3 wild-type mice and supporting gene expression patterns to confirm isolation of pure cell populations. Analysis with paired *t*-tests.

We first performed immunohistochemistry (IHC) and RNAscope on formalin-fixed paraffin-embedded mouse liver (C57Bl/6,wild-type, 1 month, *n* = 3) to spatially identify protein and RNA expression of *Vegfr1*, respectively. IHC with recombinant anti-VEGFR1 (Abcam, ab32152) identified robust expression of VEGFR1 protein in the sinusoidal regions of the liver, with little to no expression in hepatocytes (Figures 1A,A'). RNAscope with a *Vegfr1* probe (ACD, #443931) demonstrated hybridization of the probe in a sinusoidal distribution, consistent with the IHC pattern (Figures 1B,B'). These data suggested that *Vegfr1* expression was negligible in hepatocytes and strongly expressed in the sinusoidal regions of the liver, where multiple other cell types are present (i.e., non-parenchymal cells [NPCs] of the liver: endothelial cells, Kupffer cells, stellate cells, cholangiocytes, and various immune cells).

To confirm the cellular origin of *Vegfr1* expression, we performed 2-step *in vivo* liver perfusion (C57Bl/6,wild-type, 1 month, *n* = 3), as previously described (18), to generate a single cell suspension of liver cells and isolate two separate liver cell populations: hepatocytes and NPCs. After isolation of hepatocytes and NPCs, we extracted RNA from both cell populations and performed quantitative reverse-transcription PCR (qRT-PCR). We compared gene expression of *Vegfr1* and confirmed successful hepatocyte isolation by quantifying gene expression of known hepatocyte markers (Albumin [*Alb*], alpha-1-anti-trypsin [*Serpina1a*], asialoglycoprotein receptor 1 [*Asgr1*] and known NPC markers (endothelial cell: CD31 [*Pecam1*], VE-cadherin [*Cdh5*]; Kupffer cell: adhesion G protein-coupled receptor E1/EMR1 [*Adgre1*], cholangiocyte: keratin 19 [*Krt19*], leukocyte: CD45 [*Ptprc*]). Primers for each target were either previously published (19) or custom designed using Primer3 (Supplementary Table 1). Quantification was performed using the 2-ΔΔCT method (20) and groups were compared using paired t-tests. Gene expression data demonstrated that *Vegfr1* was expressed significantly more strongly in NPCs of the liver compared to hepatocytes (Figure 1C). Expression data also confirmed successful hepatocyte isolation from NPCs, which was demonstrated by enrichment of hepatocyte markers in the hepatocyte population and enrichment of NPC markers in the NPC population. Collectively, these data demonstrate that *Vegfr1* expression is negligible in hepatocytes.

## Non-hepatocyte factor: An alternative hypothesis

Our imaging and gene expression data demonstrate that *Vegfr1* is minimally expressed by mouse hepatocytes but is strongly expressed by the NPC population of mouse liver. These data suggest an alternative hepatic factor hypothesis: hepatic factor may not necessarily originate from hepatocytes. Because our field continues to have a poor understanding of the mechanism of PAVM formation and we lack data to support a single hepatic factor candidate, it is necessary to consider alternative hypotheses and alternative mechanisms.

Hepatocytes compose~80% of the liver mass but only~40% of liver cell population (21). The remainder of cell types in the liver make up the population of NPCs: endothelial cells, Kupffer cells, stellate cells, cholangiocytes, and leukocytes. Hepatocytes perform the basic cellular functions of the liver, but NPCs are critical for hepatocyte function. For example, a previous study showed that hepatocytes produce the main components of the coagulation pathway, but NPCs are necessary to activate the pathway (22). Additionally, they showed that NPC and hepatocyte crosstalk is necessary to maintain normal hepatocyte phenotype (22). Thus, hepatocytes and NPCs may work autonomously or non-autonomously to secrete hepatic factor.

The liver has diverse metabolic, hormonal, and endocrine functions (23, 24). It interacts dynamically with multiple organ systems including the mesenteric system, pancreas, adrenal glands, skeletal muscle, adipose tissue, and others (23). Thus, we should consider that the so-called hepatic factor may be influenced by factors outside the liver, or even originate from outside the liver altogether.

Rather than a singular factor secreted by hepatocytes, it is also plausible that multiple factors (or pro-factors) from various cell types in the liver may collectively perform the function of hepatic factor. A previous review highlighted that the liver secretome is diverse and includes proteins, metabolites, lipids, noncoding RNAs, extracellular vesicles, and others (24). The secretome impacts both the liver itself and distant tissues. As previously proposed by Vettukattil (8), we should consider and investigate hepatic factor candidates that include proteins, as well as miRNAs, metabolites, lipids, and other potential signaling molecules.

Finally, non-pulsatile and low shear stress pulmonary blood flow in univentricular circulation may have a larger mechanistic role than currently believed. Previous studies in HHT show that abnormal shear stress and endothelial flow response may mediate AVM pathogenesis (25–27). Thus, non-pulsatile and low shear stress flow may prime the pulmonary vasculature for PAVM formation or potentiate the function of hepatic factor.

Ultimately, we believe our data indicate that the field should consider alternative hypotheses and mechanisms if we seek to identify hepatic factor, increase our understanding of CHD-associated PAVMs, and develop therapies to improve the quality of life for our patients. Our results do not necessarily advance the field closer to identifying hepatic factor or rule out potential candidates. In fact, our data likely expand the possibilities for hepatic factor candidates and mechanisms. Based on our field's lack of progress in this domain, we believe it is necessary to consider hepatic factor candidates more broadly rather than narrowly focused on proteins secreted from hepatocytes.

In conclusion, our data demonstrate that *Vegfr1* expression is enriched in NPCs of the mouse liver and has negligible expression in mouse hepatocytes. These data exemplify our field's limited understanding of the unidentified hepatic factor. To successfully identify hepatic factor, we propose that alternative hypotheses should be considered—including that hepatic factor may not originate from hepatocytes.

## Data availability statement

The raw data supporting the conclusions of this article will be made available by the authors, without undue reservation.

## Ethics statement

The animal study was reviewed and approved by Institutional Animal Care and Use Committee, Medical College of Wisconsin.

## Author contributions

MM: performed and conceptualized experiments, interpreted data, and helped edit parts of the manuscript. AS: participated in study design, performed and conceptualized experiments, performed statistical analysis, interpreted data, and wrote and edited the manuscript. RR: participated in study design, provided intellectual input, helped conceptualize experiments, and helped edit parts of the manuscript. All authors contributed to the article and approved the submitted version.

## Funding

AS is supported by the National Institutes of Health from the National Heart, Lung, and Blood Institute (K08HL157510), the National Center for Research Resources and the National Center for Advancing Translational Sciences, National Institutes of Health (UL1TR001436), and Advancing a Healthier Wisconsin, Department of Pediatrics, and Herma Heart Institute. RR is partly supported by Children's Research Institute, Department of Pediatrics, and R61HL154254.

## Conflict of interest

The authors declare that the research was conducted in the absence of any commercial or financial relationships that could be construed as a potential conflict of interest.

## Publisher's note

All claims expressed in this article are solely those of the authors and do not necessarily represent those of their affiliated organizations, or those of the publisher, the editors and the reviewers. Any product that may be evaluated in this article, or claim that may be made by its manufacturer, is not guaranteed or endorsed by the publisher.
