# Supplementary material for: Hepatic factor may not originate from hepatocytes
Source: Front Cardiovasc Med. 2022 Sep 6;9:999315. doi: 10.3389/fcvm.2022.999315 (PMC9486074; doi:10.3389/fcvm.2022.999315)
Supplement: Supplementary file 1 [file Table_1.docx]

**Supplemental Table 1**

| Gene | Forward primer (5’-3’) | Reverse primer (5’-3’) |
| --- | --- | --- |
| *Vegfr1^#^* | AGC CCA CCT CTC TAT CCG CTG G | GGC GCT TCC GAA TCT CTA ACG |
| *Alb* | TGT CAA CCC CAA CTC TCG TG | CAG ACA CAC ACG GTT CAG GA |
| *Serpina1a* | TTC CAA CAT CTT CTT CTC CCC A | CCG ATG TTT GTG TGA GGT TGA A |
| *Asgr1* | ACA TCC CAA AAT TCC CAA CTC C | TTT CTT CCC ACA CTA CTT CCC T |
| *Pecam1* | CCG AGA GCT ACG TCA TTC CTC | TGT CAC CTT GGG CTT GGA TAC |
| *Cdh5* | ACC CAG GTT CAA GAT GCT GG | GTG TCC GGT GTC TGA TCC AG |
| *Adgre1* | CCT ATC TGT GTC TCC TGG AAC A | ATG ATA GCC AAG TTT GCC ATC C |
| *Krt19* | GCC TAC CTG AAG AAG AAC CAT G | TCT CAC TCA GGA TCT TGG CTA G |
| *Ptprc* | GTG TTC ACC TTT GCC ACT GTA T | CAC AGA CAC CCT CTC TCA CAT A |
| *Actin^#^* | AGG CCC AGA GCA AGA GAG G | TAC ATG GCT GGG GTG TTG AA |

#Primers designed by Seki et al [19]. All other primers were custom designed using Primer3.
